# Streptococcal phosphotransferase system imports unsaturated hyaluronan disaccharide derived from host extracellular matrices

**DOI:** 10.1371/journal.pone.0224753

**Published:** 2019-11-07

**Authors:** Sayoko Oiki, Yusuke Nakamichi, Yukie Maruyama, Bunzo Mikami, Kousaku Murata, Wataru Hashimoto

**Affiliations:** 1 Laboratory of Basic and Applied Molecular Biotechnology, Division of Food Science and Biotechnology, Graduate School of Agriculture, Kyoto University, Uji, Kyoto, Japan; 2 Laboratory of Food Microbiology, Department of Life Science, Faculty of Science and Engineering, Setsunan University, Neyagawa, Osaka, Japan; 3 Laboratory of Applied Structural Biology, Division of Applied Life Sciences, Graduate School of Agriculture, Kyoto University, Uji, Kyoto, Japan; University of Insubria, ITALY

## Abstract

Certain bacterial species target the polysaccharide glycosaminoglycans (GAGs) of animal extracellular matrices for colonization and/or infection. GAGs such as hyaluronan and chondroitin sulfate consist of repeating disaccharide units of uronate and amino sugar residues, and are depolymerized to unsaturated disaccharides by bacterial extracellular or cell-surface polysaccharide lyase. The disaccharides are degraded and metabolized by cytoplasmic enzymes such as unsaturated glucuronyl hydrolase, isomerase, and reductase. The genes encoding these enzymes are assembled to form a GAG genetic cluster. Here, we demonstrate the *Streptococcus agalactiae* phosphotransferase system (PTS) for import of unsaturated hyaluronan disaccharide. *S*. *agalactiae* NEM316 was found to depolymerize and assimilate hyaluronan, whereas its mutant with a disruption in the PTS genes included in the GAG cluster was unable to grow on hyaluronan, while retaining the ability to depolymerize hyaluronan. Using toluene-treated wild-type cells, the PTS activity for import of unsaturated hyaluronan disaccharide was significantly higher than that observed in the absence of the substrate. In contrast, the PTS mutant was unable to import unsaturated hyaluronan disaccharide, indicating that the corresponding PTS is the only importer of fragmented hyaluronan, which is suitable for PTS to phosphorylate the substrate at the C-6 position. This is distinct from *Streptobacillus moniliformis* ATP-binding cassette transporter for import of sulfated and non-sulfated fragmented GAGs without substrate modification. The three-dimensional structure of streptococcal EIIA, one of the PTS components, was found to contain a Rossman-fold motif by X-ray crystallization. Docking of EIIA with another component EIIB by modeling provided structural insights into the phosphate transfer mechanism. This study is the first to identify the substrate (unsaturated hyaluronan disaccharide) recognized and imported by the streptococcal PTS. The PTS and ABC transporter for import of GAGs shed light on bacterial clever colonization/infection system targeting various animal polysaccharides.

## Introduction

Extracellular matrices of all animal tissues and organs serve as physical scaffolds for cellular constituents, cell differentiation and proliferation, homeostasis, and tissue formation [1]. Glycosaminoglycans (GAGs), constituents of the matrices [2], are acidic polysaccharides consisting of repeating disaccharide units of uronate and amino sugar residues. Hyaluronan, chondroitin sulfate, heparin, and heparan sulfate are classified as GAGs based on their constituent monosaccharides, glycoside linkages, and sulfation patterns [3, 4]. Hyaluronan consists of D-glucuronate (GlcUA) and *N*-acetyl-D-glucosamine (GlcNAc), chondroitin sulfates of GlcUA and *N*-acetyl-D-galactosamine (GalNAc), and heparin and heparan sulfate of GlcUA or L-iduronate (IdoUA), and D-glucosamine (GlcN) or GlcNAc [5] (S1 Fig). The uronate and amino sugar residues in hyaluronan and chondroitin sulfate are linked by 1,3-glycoside bonds, whereas the residues in heparin and heparan sulfate are connected by 1,4-glycoside bonds. With the exception of hyaluronan, these GAGs frequently contain sulfate groups in the uronate and/or amino sugar residues, and function as protein-binding proteoglycans in extracellular matrices.

**Fig 1:**
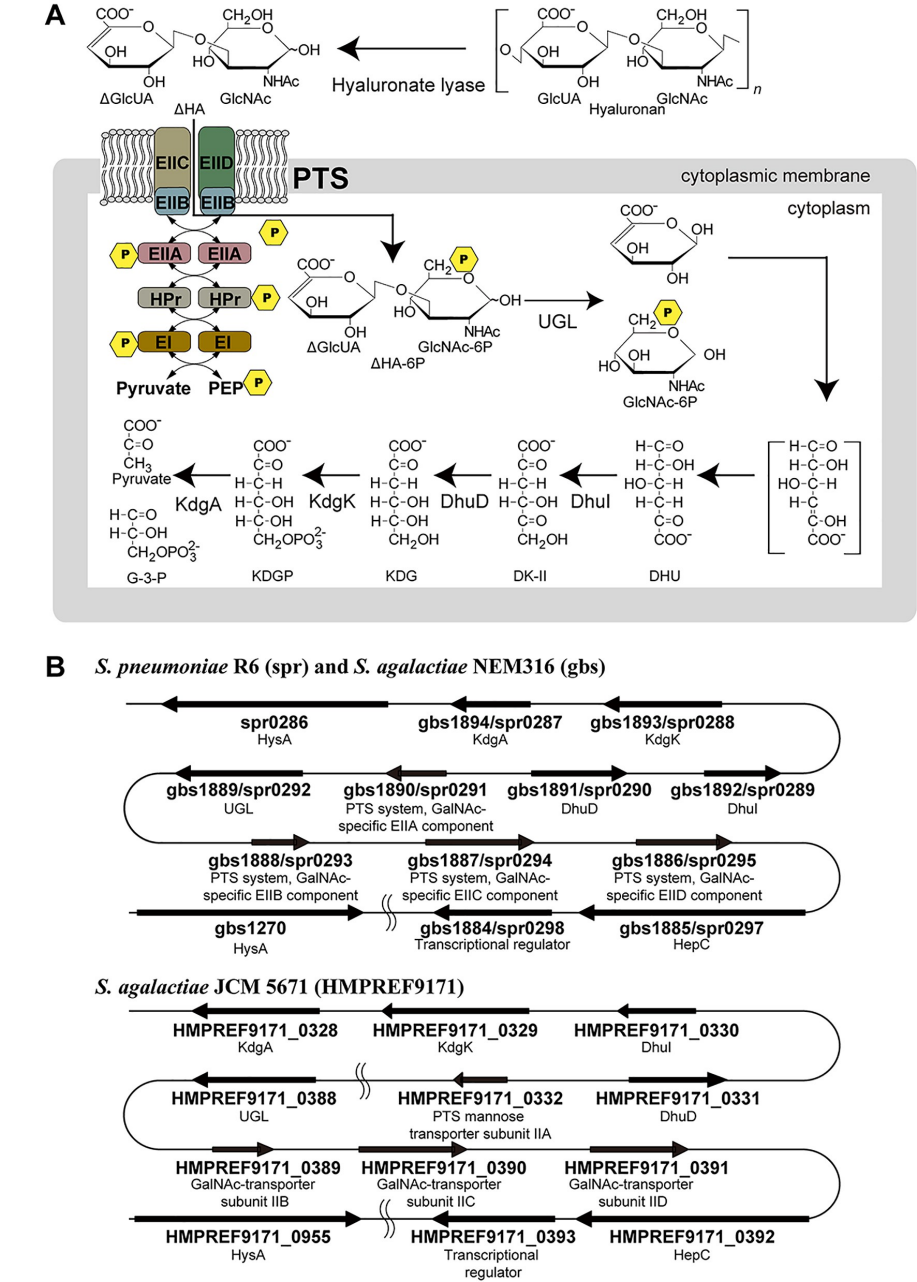
PTS import model and GAG genetic cluster. (A) *S*. *agalactiae* GAG-PTS import model. Cell-surface hyaluronate lyase (spr0286/gbs1270/HMPREF9171_0955) depolymerizes hyaluronan and the resulting unsaturated hyaluronan disaccharides are incorporated into the cytoplasm by GAG-PTS (spr0291-0293-0294-0295/gbs1886-1887-1888-1890/HMPREF9171_0332-0389-0390-0391). During the import process, a phosphate group is transferred to the substrate. After import has been achieved, unsaturated glucuronyl hydrolase (UGL) (spr0292/gbs1889/HMPREF9171_0388) degrades the disaccharides to monosaccharides. The resulting unsaturated uronate is non-enzymatically converted to 4-deoxy-L-*threo*-5-hexosulose-uronate (DHU). DHU is metabolized to 3-deoxy-D-*glycero*-2,5-hexodiulosonate (DK-II) by 4-deoxy-L-*threo*-5-hexosulose-uronate ketol-isomerase (DhuI) (spr0289/gbs1892/HMPREF9171_0330). DK-II is then metabolized to 2-keto-3-deoxy-D-gluconate (KDG) by 2-keto-3-deoxy-D-gluconate dehydrogenase (DhuD) (spr0290/gbs1891/HMPREF9171_0331). KDG is converted to pyruvate and glyceraldehyde-3-phosphate (G-3-P) via 2-keto-3-deoxy-6-phosphogluconate (KDGP), through successive reactions catalyzed by 2-keto-3-deoxygluconate kinase (KdgK) (spr0288/gbs1893/HMPREF9171_0329) and 2-keto-3-deoxy-6-phosphogluconate aldolase (KdgA) (spr0287/gbs1894/HMPREF9171_0328). (B) Upper, GAG genetic clusters in the genomes of *S*. *pneumoniae* R6 (spr) and *S*. *agalactiae* NEM316 (gbs); and lower, GAG genetic cluster in the genome of *S*. *agalactiae* JCM 5671 (HMPREF9171). The GAG genetic cluster in the *S*. *agalactiae* JCM 5671 is divided into two segments by the insertion of 55 genes.

Some bacteria including staphylococci and streptococci target animal GAGs for colonization and/or infection [6]. GAGs are degraded by two chemically distinct enzymatic mechanisms, hydrolases and lyases [7]. Hydrolases cleave the glycoside bonds between the glycosyl oxygen and the anomeric carbon atom by addition of a water. In contrast, lyases recognize the uronate residues and cleave the glycoside bonds through a β-elimination reaction, resulting in producing unsaturated disaccharides with C = C double bonds at the nonreducing terminus of the uronate residues. Streptococci are known to invade host cells by the depolymerization of hyaluronan using cell-surface hyaluronate lyase through the β-elimination reaction [8–12] (Fig 1A). Our previous reports indicate that the resulting unsaturated GAG disaccharides are degraded in the cytoplasm by unsaturated glucuronyl hydrolase (UGL) into monosaccharides (unsaturated uronate and amino sugar) through the hydration of the C = C double bonds [13–15]. Moreover, unsaturated uronate was shown to metabolize to pyruvate and glyceraldehyde-3-phosphate through successive reactions catalyzed by isomerase (DhuI), NADH-dependent reductase (DhuD), kinase (KdgK), and aldolase (KdgA) [16], while GlcUA is known to be metabolized in different pathways (S2 Fig) [17]. Although the bacterial import of GAGs was poorly understood, we have recently identified a solute-binding protein-dependent ATP-binding cassette (ABC) transporter in a pathogenic *Streptobacillus moniliformis* as the importer of the fragmented GAGs [18].

Streptococci such as *Streptococcus pneumoniae*, *Streptococcus pyogenes*, and *Streptococcus agalactiae*, are classified into three groups based on their hemolytic activity [19]. In *S*. *pneumoniae* genomes, enzymes for the depolymerization, degradation, and metabolism of GAGs are encoded together with a putative phosphotransferase system (PTS), a sugar import system specific for bacteria [20]. The genes encoding hyaluronate lyase, UGL, DhuI, DhuD, and the PTS are assembled to form a GAG genetic cluster (Fig 1B). The similar genetic cluster is also included in the genome of *S*. *pyogenes* and *S*. *agalactiae*.

PTS is composed of Enzyme I (EI), histidine-containing phosphocarrier protein (HPr), and Enzyme II (EII), which has multiple hetero-subunits (EIIA, EIIB, EIIC, and EIID) [21]. Cytoplasmic EI and HPr proteins are common to all PTSs in bacterial cells and nonspecifically recognize sugar substrates, whereas various EIIs are substrate-specific and consist of cell membrane and cytoplasmic domains. Only EII (EIIA, EIIB, EIIC, and EIID) genes are found in the streptococcal GAG cluster, while EI and HPr genes are also located on the bacterial genome. Mechanistically, PTSs import sugar by phosphorylating the substrate at the C-6 position through successive phosphotransfer reactions from a phosphate donor (phosphoenolpyruvate) mediated by EI, HPr, and EII [20]. A large number of GAGs (with the exception of hyaluronan) are frequently sulfated at the C-6 position [22]. Unsaturated GAG disaccharides with a sulfate group at C-6 are unsuitable as PTS substrates due to the lack of phosphorylation. Indeed, after disruption of the EI gene, *Salmonella typhimurium* still grows on sugars such as GlcUA and glucose-6-phosphate, indicating that sugars with carboxyl or phosphate group at their C-6 position are imported by other transport systems distinct from PTSs [23]. Despite the identification of more than twenty sugars that are imported by PTSs, none are modified at the C-6 position [24].

The PTS encoded in the GAG genetic cluster (GAG-PTS) is thought to import depolymerized hyaluronan because the presence of hyaluronan leads to an increase in the expression of the *S*. *agalactiae* GAG-PTS gene [25]. Marion *et al*. have previously shown that the GAG-PTS, in conjunction with hyaluronate lyase and UGL, is essential for the growth of *S*. *pneumoniae* when hyaluronan is used as the sole carbon source [26]. GAG-PTS mutation has been shown to reduce the ability of the bacteria to colonize mouse upper respiratory tracts. However, the substrate of the GAG-PTS remains to be identified. This study focused on the role of the *S*. *agalactiae* GAG-PTS in the import of unsaturated hyaluronan disaccharides as the substrate. Moreover, structure determination of *S*. *agalactiae* GAG-PTS EIIA by X-ray crystallography and docking of the EIIA with EIIB provided structural insights into the phosphate transfer mechanism.

## Materials and methods

### Materials

Hyaluronan sodium salt was purchased from Sigma Aldrich. Sodium salts of chondroitin sulfates A and C were obtained from Wako Pure Chemical Industries, and heparin sodium salt from Nacalai Tesque. A thermosensitive suicide vector, pSET4s, was kindly provided by Dr. Takamatsu (National Agriculture and Food Research Organization).

### Microorganisms and culture conditions

*S*. *agalactiae* NEM316 (ATCC 12403) was purchased from Institute Pasteur, and *S*. *agalactiae* JCM 5671 (ATCC 13813) from Riken BioResource Center. *S*. *agalactiae* cells were statically grown at 37°C under 5% CO_2_ in 3.7% brain heart infusion medium (BD Bacto) or 0.8% nutrient medium (0.3% beef extract and 0.5% peptone) (Difco), supplemented with 20% horse serum for 16–24 h. To investigate hyaluronan assimilation by *S*. *agalactiae*, hyaluronan-containing minimal medium was prepared as described previously [26, 27]. Briefly, streptococcal cells in logarithmic phase of growth were inoculated (to an optical density of 0.01 at 600 nm; OD_600_) into minimal medium consisting of 0.44 g/l KH_2_PO_4_, 0.3 g/l K_2_HPO_4_, 3.15 g/l Na_2_HPO_4_, 2.05 g/l NaH_2_PO_4_, 0.225 g/l sodium citrate, 6 g/l sodium acetate, 0.6 g/l (NH_4_)_2_SO_4_, 0.2 g/l MgSO_4_, 10 mg/l NaCl, 10 mg/l FeSO_4_, 10 mg/l MnSO_4_, 0.4 mg/l riboflavin, 0.01 mg/l biotin, 0.1 mg/l folate, 0.8 mg/l pantothenate, 0.4 mg/l thiamine, 2 mg/l nicotinamide, 0.8 mg/l pyridoxamine, 0.1 mg/l *p*-aminobenzoate, 5 mg/l Gln, 300 mg/l Glu, 110 mg/l Lys, 100 mg/l Asp, 100 mg/l Ile, 100 mg/l Leu, 100 mg/l Met, 100 mg/l Ser, 100 mg/l Phe, 100 mg/l Thr, 100 mg/l Val, 200 mg/l Ala, 200 mg/l Arg, 200 mg/l Cys, 200 mg/l His, 200 mg/l Gly, 400 mg/l Pro, 200 mg/l Trp, 200 mg/l Tyr, 35 mg/l adenine and 30 mg/l uracil, in the presence of 0.5% hyaluronan or 2% glucose, or in the absence of sugar substrate. The bacterial cells were grown at 37°C and turbidity monitored periodically.

*Escherichia coli* DH5α cells harboring plasmids were cultured at 37°C in Luria-Bertani (LB) medium containing 100 μg/ml sodium ampicillin. For the expression of recombinant proteins, *E*. *coli* BL21(DE3) cells harboring plasmids were cultured at 30°C in LB medium containing 100 μg/ml sodium ampicillin to an OD_600_ of 0.3–0.7, followed by the addition of isopropyl-β-D-thiogalactopyranoside to a final concentration of 0.1 mM, and further incubation at 16°C for 2 days.

### Halo detection for GAG degradation

Halo detection method was used to investigate the GAG-degrading ability of *S*. *agalactiae*. The bacterial cells were grown on plates containing 0.2% dialyzed GAG (hyaluronan, chondroitin sulfate A, C, or heparin), 0.8% nutrient medium, 20% horse serum, and 1% bovine serum albumin (BSA) solidified with 1% agar. When sufficient bacterial growth was achieved, the addition of 2 M acetic acid (1 ml) to the plates resulted in the formation of a white precipitate due to the interaction of GAGs and BSA; areas containing degraded GAGs appear as clear zones or “halos”.

### Construction of an overexpression system

An overexpression system for *S*. *agalactiae* hyaluronate lyase was constructed in *E*. *coli* as a source of enzymes for the preparation of unsaturated hyaluronan disaccharide required for the PTS import assay. To clone the gbs1270 gene that encodes hyaluronate lyase, polymerase chain reaction (PCR) was conducted on 10 μl of reaction mixture consisting of 0.2 U of KOD Plus Neo polymerase (Toyobo), *S*. *agalactiae* cells as a template, 0.3 pmol of each of forward and reverse primers (Table 1, gbs1270_F and gbs1270_R), 2 nmol of dNTPs, 10 nmol of MgCl_2_, 0.5 μl of dimethyl sulfoxide, and the commercial reaction buffer supplied with KOD Plus Neo polymerase. PCR conditions were as follows: 94°C for 2 min followed by 30 cycles of 98°C for 10 s, 35°C for 30 s, and 68°C for 2 min. The PCR product was ligated to *Hin*cII-digested pUC119 (Takara Bio) using Ligation High Ver. 2 (Toyobo), and the resulting plasmid was digested with *Nco*I and *Xho*I to isolate the gbs1270 gene. The gene fragment was confirmed to encode the correct gbs1270 by DNA sequencing [28]. The *Nco*I and *Xho*I-digested gbs1270 gene was ligated into *Nco*I and *Xho*I-digested pET21d (Novagen), and *E*. *coli* BL21(DE3) host cells were transformed with the resulting plasmid, pET21d-gbs1270. An overexpression system of *S*. *agalactiae* EIIA^ΔHA^ (EIIA for unsaturated hyaluronan disaccharide; ΔHA) was also constructed in *E*. *coli* for X-ray crystallography. To clone the gbs1890 gene encoding EIIA^ΔHA^, PCR was performed using primers specific for EIIA^ΔHA^ (Table 1, gbs1890_F and gbs1890_R) as described above. The gene fragment was ligated into *Nde*I and *Xho*I-digested pET21b (Novagen), and *E*. *coli* BL21(DE3) host cells were transformed with the plasmid pET21b-gbs1890.

**Table 1:** Primers used in this study.

Primer	Sequence
gbs1270_F	5’-GGCCATGGAAATCAAAAAGAAACATCGTATTATG-3’
gbs1270_R	5’- CCCCTCGAGGATAGCTAATTGGTCTGTTTTTGTCATG-3’
gbs1890_F	5’-GGCATATGATAAAAATTATTATTGTAGCACACGGC-3’
gbs1890_R	5’-GGCTCGAGAATGCCTCCCTCAAAAGTTGCTTCTGCAGT-3’
gbs1886-1887-1888_F	5’- ATGGCAGCAGGACCAAATATTG -3’
gbs1886-1887-1888_R	5’-TTAAGCTAAAATACCTAACCAGCTACCAAG-3’
gbs1886-1887-1888_invF	5’- GTATCGGTGATTCTTTATCACAATTTTGC -3’
gbs1886-1887-1888_invR	5’- TCCAACTAAATATAATCTAAAATATTAACTTCCACAGC -3’
Km^r^_F	5’- CCTGGCCAGGGGGAAAGCCACGTTGTGTCTCAAAA -3’
Km^r^_R	5’- CCTGGCCAGGGGGCGCTGAGGTCTGCCTCGTGAAG -3’

### Construction of the PTS mutant

The GAG-PTS mutant was constructed using a kanamycin-resistant gene (Km^r^) and the thermosensitive suicide vector, pSET4s. The gbs1886-1887-1888 operon gene coding for GAG-PTS EIID, EIIC, and EIIB was amplified by PCR using *S*. *agalactiae* cells as a template and primers (Table 1, gbs1886-1887-1888_F and gbs1886-1887-1888_R), and the PCR product was ligated with *Hin*cII-digested pSET4s. Using the pSET4s-gbs1886-1887-1888 plasmid as a template and primers (Table 1, gbs1886-1887-1888_invF and gbs1886-1887-1888_invR), inverse PCR was conducted to amplify linear PCR product to remove the GAG-PTS operon gene excepting the both ends of 500 bp for homologous recombination. The lineared product of inverse PCR was ligated with pUC4K-derived Km^r^ amplified by PCR using primers (Table 1, Km^r^_F and Km^r^_R). The resulting plasmid was designed as pSET4s-gbs1886-1887-1888::Km^r^.

To transform the pSET4s-gbs1886-1887-1888::Km^r^ plasmid into the streptococcal cells, electrotransformation was conducted as previously described, but with a slight modification [29]. Briefly, *S*. *agalactiae* NEM316 was grown in 50 ml brain heart infusion medium containing 0.4% glucose to an OD_600_ of 0.3, harvested by centrifugation at 2,610 *g* at 4°C for 10 min, and washed three times with 15 ml of 10% cold glycerol. The washed cells were suspended in 1 ml of 20% cold glycerol and aliquoted into 50 μl samples. Following the addition of 1 μg of the plasmid (1 μg/μl) and incubation on ice for 1 min, the competent cells were transferred to a cold electroporation cuvette with a 0.1 cm gap (Bio-Rad). The cuvette was set to MicroPulser (Bio-Rad) and pulsed as follows: field strength, 1.8 kV; capacitor, 10 μF; and resistor, 600 Ω. Brain heart infusion medium containing 10% glycerol (1 ml) was immediately and gently added to the cuvette. After incubation at 28°C for 1 h, the electroporated cells were spread on a brain heart infusion plate containing 250 μg/ml spectinomycin, and further incubated at 28°C for 3 days to obtain a spectinomycin-resistant transformant. The single crossover mutant cells were transferred to medium without spectinomycin and subcultured repeatedly at 37°C. A spectinomycin-sensitive and kanamycin-resistant (500 μg/ml) single colony was considered to be double crossover mutant. PCR was conducted to confirm the gene disruption.

### Protein purification

Recombinant *E*. *coli* cells were harvested by centrifugation at 6,700 *g* at 4°C for 10 min and suspended in 20 mM Tris (hydroxymethyl) aminomethane-hydrochloride (Tris-HCl), pH 7.5. The cell suspension was ultrasonicated (Insonator Model 201M, Kubota) at 0°C and 9 kHz for 10 min, and subjected to centrifugation at 20,000 *g* at 4°C for 20 min. The supernatant cell extract was then used in subsequent experiments. The BL21(DE3)/pET21d-gbs1270 cell extract was used as a source of hyaluronate lyase for the preparation of unsaturated hyaluronan disaccharide. EIIA^ΔHA^ was purified from BL21(DE3)/pET21b-gbs1890 cell extract using metal affinity [TALON (Clontech)] and gel filtration chromatography [Sephacryl S-200 (GE Healthcare)]. After the confirmation of protein purity by sodium dodecyl sulfate-polyacrylamide gel electrophoresis (SDS-PAGE), the purified protein was dialyzed against 20 mM Tris-HCl (pH 7.5).

### Preparation of unsaturated hyaluronan disaccharide

To investigate GAG-PTS import activity, unsaturated hyaluronan disaccharide was prepared using recombinant hyaluronate lyase. A reaction mixture containing BL21(DE3)/pET21d-gbs1270 cell extract, 0.2% hyaluronan, and 20 mM Tris-HCl (pH 7.5) was incubated at 30°C for 2 days. The mixture was then boiled to stop the reaction and centrifuged at 20,000 *g* for 20 min to remove aggregated proteins. The resulting supernatant was concentrated by freeze-drying and subjected to gel filtration chromatography [Superdex Peptide 10/300 GL (GE Healthcare)]. The eluted fractions containing unsaturated hyaluronan disaccharide were identified by monitoring the absorbance (235 nm) from the C = C double bonds of the disaccharide. To confirm the presence of unsaturated hyaluronan disaccharide, pooled fractions were subjected to thin-layer chromatography (TLC) using a solvent system of 1-butanol:acetic acid:water (3:2:2, v:v:v), and hyaluronan breakdown products were visualized by heating the TLC plates [silica gel 60 F_254_ (Merck)] at 130°C for 5 min after spraying with ethanol containing 10% sulfuric acid. The final disaccharide preparation was freeze-dried and dissolved in sterilized water to a final concentration of 200 mM calculated from the absorption coefficient.

### PTS assay

The import of unsaturated hyaluronan disaccharides into *S*. *agalactiae* via the GAG-PTS was evaluated by quantifying the pyruvate produced from phosphoenolpyruvate during the import process, as described previously but with a few modifications [30, 31]. The reaction mixture contained cells whose cell-surface layer had been permeabilized with toluene, phosphoenolpyruvate, disaccharides, NADH, and L-lactate dehydrogenase from rabbit muscle (Oriental Yeast). The reaction was monitored through measurements of absorbance at 340 nm to determine levels of NADH oxidation resulting from the production of lactate from the pyruvate generated by the PTS process. Briefly, *S*. *agalactiae* wild-type and GAG-PTS mutant cells were grown at 37°C under 5% CO_2_ to exponential phase in 0.8% nutrient medium and 20% horse serum, in the presence or absence of 0.2% dialyzed hyaluronan, and then harvested by centrifugation at 2,610 *g* at 4°C for 5 min. The cells were washed twice with 5 mM MgCl_2_ and 0.1 M potassium phosphate buffer (KPB); pH 7.2, and suspended in 1 ml of the same buffer containing 50 μl of acetone:toluene (9:1, v:v) by vortexing twice for 2 min. The reaction mixture containing toluene-treated cells, 10 mM sugar, 0.1 mM NADH, 0.023 mg/ml L-lactate dehydrogenase, 10 mM NaF, 5 mM MgCl_2_, and 0.1 M KPB (pH 7.5) was incubated at 37°C for 5 min, and phosphoenolpyruvate then added to a concentration of 5 mM. The reaction was monitored by measuring the decrease in absorbance at 340 nm. No decrease in absorbance at 340 nm was detected in the absence of phosphoenolpyruvate. The protein concentration of toluene-treated cells was determined using the bicinchoninic acid (BCA) assay [32]. The PTS import activity value was calculated as the amount of pyruvate produced (nmol/min/mg).

### X-ray crystallography

To determine the three-dimensional structure of *S*. *agalactiae* EIIA^ΔHA^, the purified protein was concentrated to 9.24 mg/ml and crystallized using the sitting drop vapor diffusion method. The purified EIIA^ΔHA^ (1 μl) was mixed with an equal volume of a reservoir solution consisting of 20% (w/v) polyethylene glycol (PEG) 3,350 and 0.2 M sodium thiocyanate (pH 6.9), and incubated at 20°C. The crystal was picked up from the drop with a nylon loop, soaked in reservoir solution containing 20% glycerol as a cryoprotectant, and instantaneously frozen in liquid nitrogen. X-ray diffraction data were collected at the BL38B1 station of SPring-8 (Hyogo, Japan). The data were indexed, integrated, and scaled using *HKL2000* software [33]. The structure was determined through the molecular replacement method using *Molrep* in the *CCP4* software package and *E*. *coli* PTS EIIA (PDB ID, 1PDO) for mannose import [34]. Structure refinement was conducted with *phenix refine* in *PHENIX* software [35]. The model was refined manually with *winCoot* software [36]. Protein structures were prepared using the *PyMOL* [37].

## Results

### Degradation of GAGs by *S*. *agalactiae*

**Fig 2:**
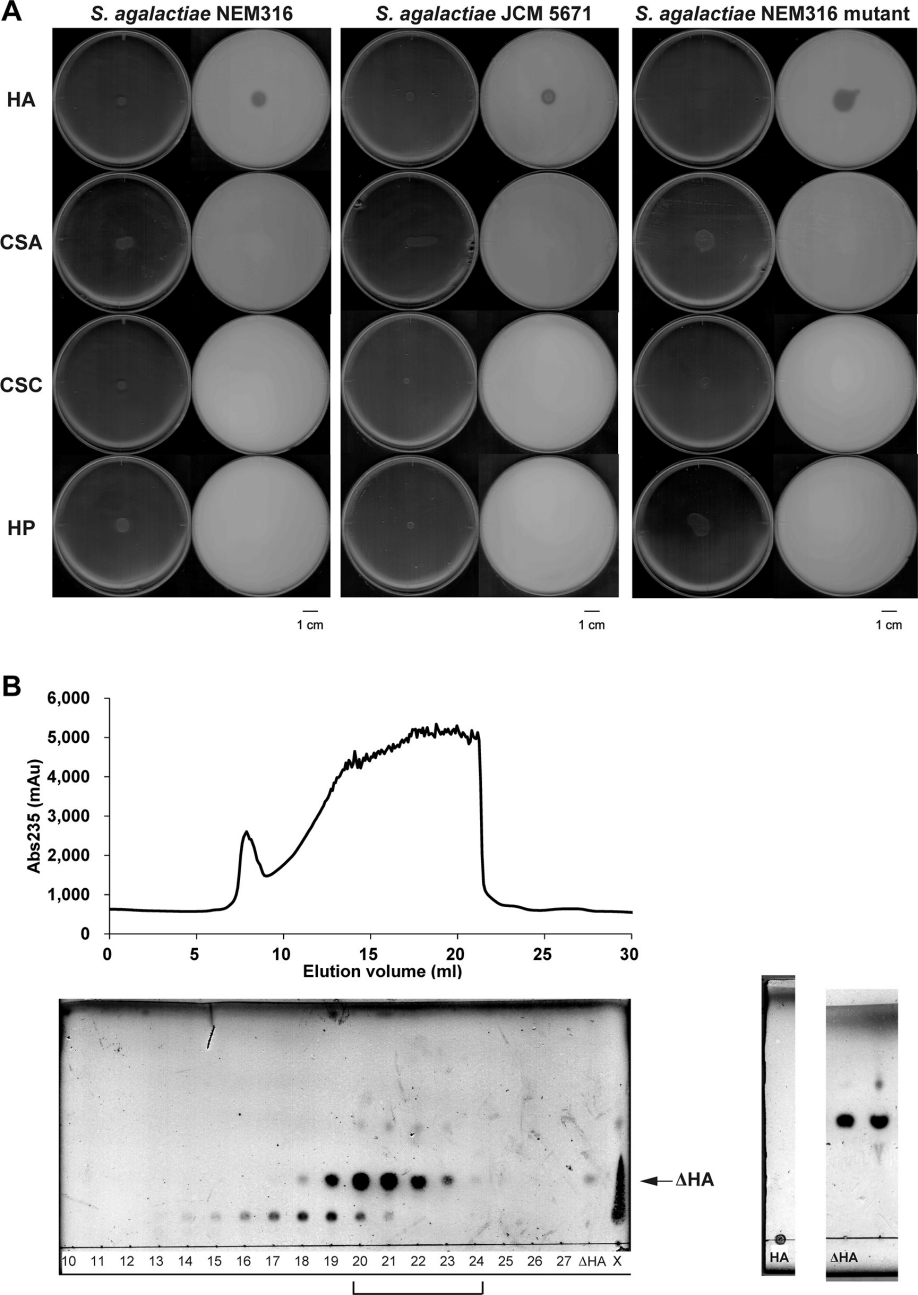
Degradation of GAGs by *S*. *agalactiae*. (A) Degradation of GAGs by *S*. *agalactiae* NEM316, *S*. *agalactiae* JCM 5671, and *S*. *agalactiae* NEM316 GAG-PTS mutant. The left and right plates in each panel are images taken before and after the addition of acetic acid, respectively. Plates contained hyaluronan (HA), chondroitin sulfate A (CSA), chondroitin sulfate C (CSC), or heparin (HP). (B) Preparation of unsaturated hyaluronan disaccharide. Shown are the elution profiles of unsaturated hyaluronan disaccharide during gel filtration chromatography (upper), and TLC profiles of fractions from gel filtration chromatography (lower, left). Numbers denote elution volume (ml). X represents a sample of the reaction mixture before gel filtration chromatography. Hyaluronan (HA) without degradation showed a spot at the original point (lower, center). TLC profiles of the mixture of collected fractions are also shown (lower, right). The mixture (lane, right) was found to contain a saccharide as a main product that corresponded to the standard unsaturated hyaluronan disaccharide (ΔHA) (lane, left). These profiles are not an image cropped from different parts of the same TLC plate or from different TLC plates.

As *S*. *agalactiae* produces hyaluronate lyase that depolymerizes both hyaluronan and unsulfated region of chondroitin sulfate [38], the halo plate method was used to investigate streptococcal GAG degradation (Fig 2A). GAGs and BSA contained in the plates form the white precipitate derived from the aggregation upon the addition of acetic acid, while degraded GAGs by bacterial cells appear to be clear as “halo”. Chondroitin sulfate is classified into chondroitin sulfates A, B, and C, based on the position of the sulfate group [39]. Chondroitin sulfate C is sulfated at the C-6 position of GalNAc, whereas chondroitin sulfates A and B are sulfated at the C-4 position. The repeating units of chondroitin sulfates A, B, and C are GlcUA-GalNAc4S (GalNAc with a sulfate group at the C-4 position), IdoUA-GalNAc4S, and GlcUA-GalNAc6S (GalNAc with a sulfate group at the C-6 position), respectively [40]. Plates containing the brain heart infusion necessary for streptococcal growth were unsuitable for formation of the white precipitate by GAGs and BSA. Accordingly, nutrient medium and horse serum were used as alternatives for halo plate analysis.

In addition to *S*. *agalactiae* NEM316 (Fig 1B, upper), *S*. *agalactiae* JCM 5671 (which contains the GAG genetic cluster; Fig 1B, lower) was selected to represent a typical strain that is able to degrade GAG. Moreover, the function of the GAG-PTS encoded in the GAG genetic cluster was characterized through the construction of a NEM316 mutant strain by replacing the GAG-PTS gene segment (a set of EIIB, EIIC, and EIID genes) with Km^r^, which is referred to PTS mutant in this study (S3 Fig); the degrading ability of this GAG-PTS mutant was then assessed.

Although the GAG genetic cluster of *S*. *agalactiae* JCM 5671 is divided into two segments by the insertion of 55 genes between the HMPREF9171_0332 gene encoding the GAG-PTS EIIA and the HMPREF9171_0388 gene encoding UGL (Fig 1B, lower), similar to strain NEM 316, strain JCM 5671 also produced clear halos on plates containing hyaluronan (Fig 2A). However, halos were not observed on plates containing chondroitin sulfate A or C, or heparin. This indicates that *S*. *agalactiae* is active against hyaluronan, but not the other three GAGs. The lack of chondroitin sulfate A and C degradation was probably due to a low level of bacterial lyase activity toward chondroitin sulfates. As expected, the GAG-PTS mutant exhibited a halo on hyaluronan-containing plates at same level with the wild-type (but not on those containing the other GAGs), suggesting that the GAG-PTS has no influence on the degradation of hyaluronan.

### Assimilation of hyaluronan by *S*. *agalactiae*

**Fig 3:**
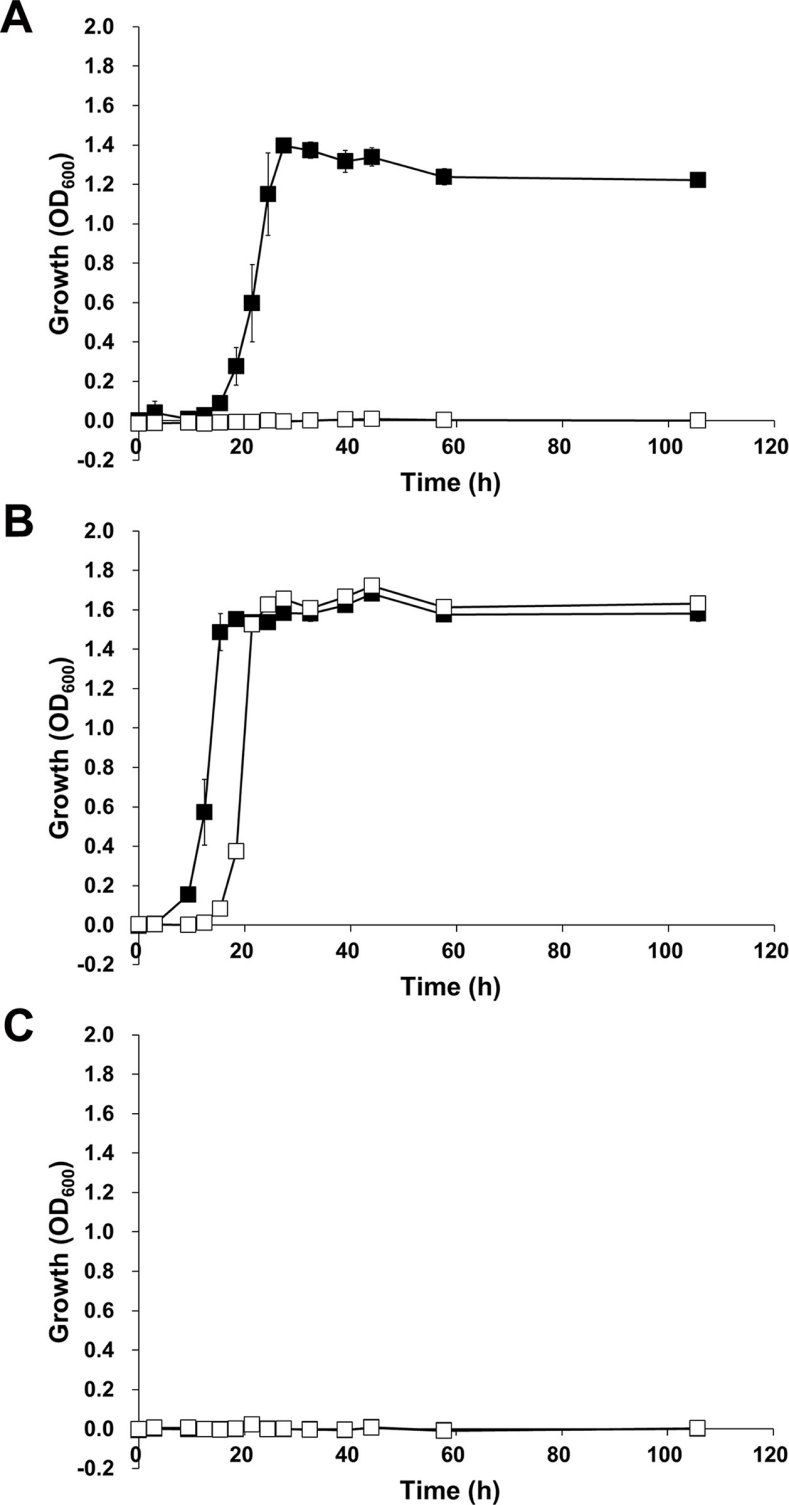
Growth of *S*. *agalactiae* in the presence of hyaluronan. Wild-type (closed) and the GAG-PTS mutant (open) in minimal medium containing hyaluronan (A), glucose (B), or no saccharide (C). Each measurement represents the mean of three individual experiments (means ± standard deviations). Doubling times at the exponential growth phase of wild-type in hyaluronan medium, 2.09 h; wild-type in glucose medium, 1.72 h; and the GAG-PTS mutant in glucose medium, 1.69 h.

*S*. *agalactiae* GD201008-001 has been shown to use hyaluronan as a sole carbon source for growth [41]. In addition, a *S*. *pneumoniae* mutant with a disruption of the GAG-PTS genes in the GAG genetic cluster was unable to assimilate hyaluronan [26]. Based on these observations, the hyaluronan assimilation of *S*. *agalactiae* NEM316 (Fig 1B, upper) and its GAG-PTS mutant was investigated using hyaluronan-containing minimal medium (Fig 3). *S*. *agalactiae* (wild-type) was found to grow on hyaluronan (Fig 3A) or glucose (Fig 3B), whereas no growth was apparent on minimal medium that lacked saccharide (Fig 3C). In contrast, the GAG-PTS mutant was unable to grow in the hyaluronan-containing minimal medium, indicating that the GAG-PTS encoded in the GAG genetic cluster is crucial for the assimilation of hyaluronan in *S*. *agalactiae*. In addition, the growth of wild-type cells on hyaluronan-containing media was higher than that observed in the absence of hyaluronan, whereas the growth of the GAG-PTS mutant cells was unaffected (S4 Fig).

As *S*. *agalactiae* was found to degrade and assimilate hyaluronan, GAG-PTS activity was investigated by the preparation of unsaturated hyaluronan disaccharide using recombinant bacterial hyaluronate lyase (Fig 2B). An overexpression system for *S*. *agalactiae* hyaluronate lyase was constructed in *E*. *coli*, and the cell extract was used to treat hyaluronan. The reaction product was then purified by gel filtration chromatography (Fig 2B, upper). The eluted fractions were subjected to TLC (Fig 2B, lower), and the fractions containing unsaturated hyaluronan disaccharide at an elution volume of 20–24 ml were collected, concentrated, and used as the substrate in the PTS assay.

### Import of unsaturated hyaluronan disaccharide by *S*. *agalactiae* PTS

**Fig 4:**
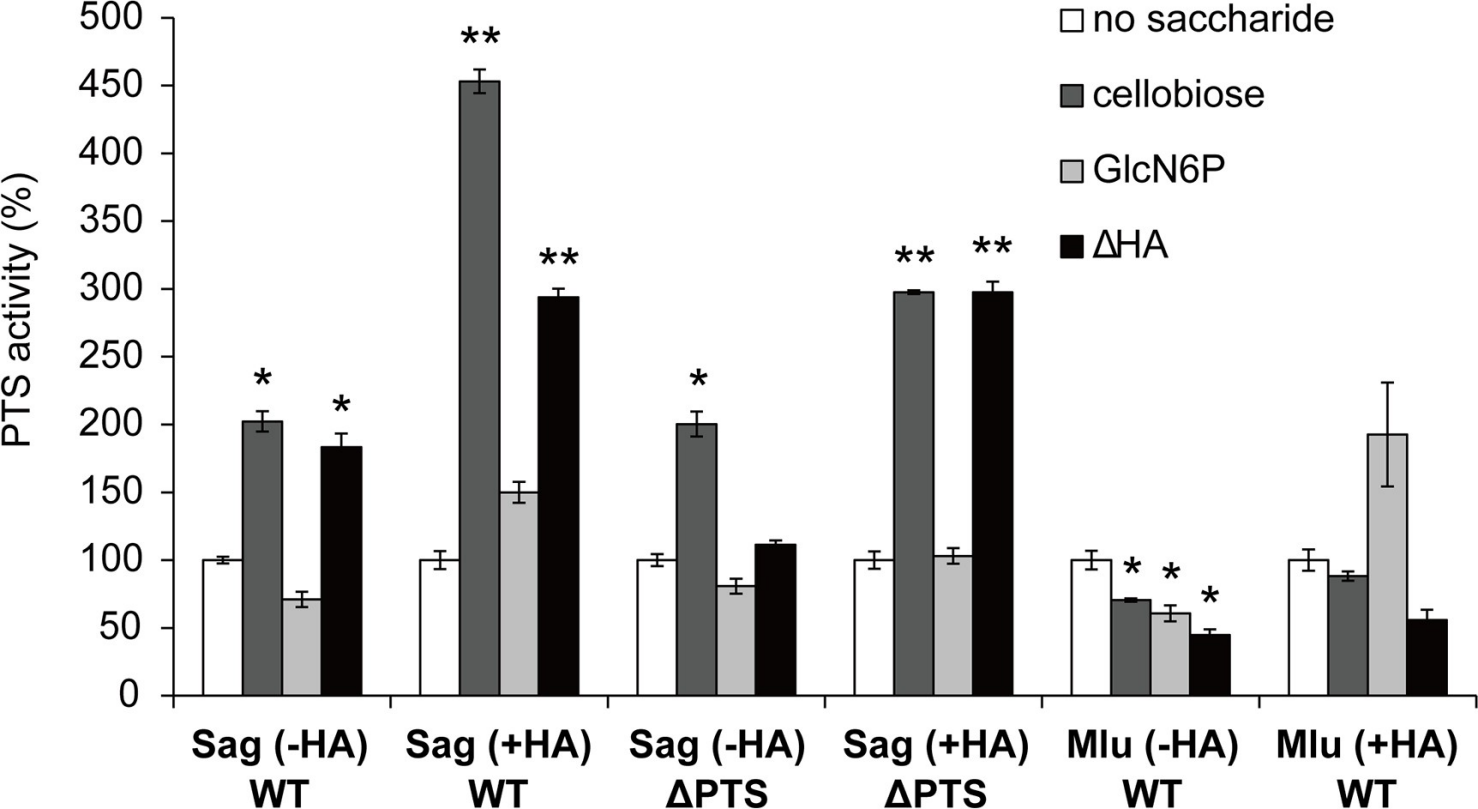
Import of unsaturated hyaluronan disaccharide by *S*. *agalactiae* GAG-PTS. Levels of GAG-PTS import into *S*. *agalactiae* and *M*. *luteus* grown in the absence or presence of hyaluronan. *S*. *agalactiae* wild-type cells grown in the absence of hyaluronan, Sag (-HA) WT; *S*. *agalactiae* wild-type cells grown in the presence of hyaluronan, Sag (+HA) WT; *S*. *agalactiae* GAG-PTS mutant cells grown in the absence of hyaluronan, Sag (-HA) ΔPTS; *S*. *agalactiae* GAG-PTS mutant cells grown in the presence of hyaluronan, Sag (+HA) ΔPTS; *M*. *luteus* wild-type cells grown in the absence of hyaluronan, Mlu (-HA) WT; and *M*. *luteus* wild-type grown in the presence of hyaluronan, Mlu (+HA) WT. No saccharide, white; positive control (cellobiose), dark gray; negative control (GlcN6P), light gray; and unsaturated hyaluronan disaccharide (ΔHA), black. Each measurement represents the mean of three individual experiments (means ± standard deviations). Significant differences from the control were statistically determined using Student’s t-test (**p < 0.01; *p < 0.05).

To demonstrate the GAG-PTS-dependent import of unsaturated hyaluronan disaccharide in *S*. *agalactiae* NEM316, GAG-PTS-induced pyruvate production from phosphoenolpyruvate was measured using bacterial cells permeabilized by treatment with toluene (Fig 4). As *S*. *pneumoniae* has previously been shown to incorporate cellobiose via a PTS [42] and include PTS (EIIA, EIIB, EIIC, and EIID) genes for import of cellobiose in the genome, cellobiose was used as a positive control. In contrast, D-glucosamine-6-phosphate (GlcN6P) was used as a negative control because phosphorylation at the C-6 position renders it an unsuitable PTS substrate. Gram-positive *Micrococcus luteus*, which lacks both the GAG genetic cluster and PTS genes, was also used as a negative control. Because of an increase in the expression of the *S*. *agalactiae* GAG-PTS gene in the presence of hyaluronan, bacterial cells grown in the presence of hyaluronan were also used in this assay.

The growth of *S*. *agalactiae* both in the presence and absence of hyaluronan led to an increase in the PTS import of cellobiose (compared to the basal activity measured in the absence of the sugar substrate). This indicates that the bacterial PTS is promoting the uptake of cellobiose into the cell. In contrast, *S*. *agalactiae* exhibited no enhanced PTS activity using GlcN6P as a substrate, regardless of the presence of hyaluronan, i.e. no significant difference was observed between the cells grown in the presence and absence of hyaluronan. These results suggest that permeabilized *S*. *agalactiae* cells are functionally active, and the assay used is a reliable indicator of PTS activity. The bacterial cells exhibited a higher level of PTS-mediated cellobiose import when grown in the presence of hyaluronan. Furthermore, *M*. *luteus* exhibited comparable levels of PTS import of cellobiose as the basal controls; this reflects the lack of a cellobiose PTS in *M*. *luteus*. Limited PTS import of GlcN6P was observed in *M*. *luteus* cells, which is in agreement with the results from *S*. *agalactiae*.

The levels of the GAG-PTS import of unsaturated hyaluronan disaccharide into *S*. *agalactiae* grown in the absence and presence of hyaluronan were approximately 1.8 and 2.9 times higher than basal levels, respectively. These findings represent a significant difference between the GAG-PTS import and basal activity, especially in cells grown in the presence of hyaluronan. On the other hand, the GAG-PTS mutant grown in the absence of hyaluronan showed similar levels of GAG-PTS import of unsaturated hyaluronan disaccharide as the basal controls. This indicates that the mutant lacks the ability to import unsaturated hyaluronan disaccharide. These results clearly demonstrate that *S*. *agalactiae* imports unsaturated hyaluronan disaccharide using the GAG-PTS encoded in the GAG genetic cluster. Unexpectedly, the GAG-PTS mutant grown in the presence of hyaluronan showed an increased PTS activity compared with that in the absence of hyaluronan.

### Structure determination of *S*. *agalactiae* EIIA^ΔHA^

X-ray crystallography of EIIA^ΔHA^ was performed as a first step toward determining the overall structure of the *S*. *agalactiae* GAG-PTS complex (EIIABCD) for the import of unsaturated hyaluronan disaccharide. Recombinant purified EIIA^ΔHA^ protein was crystallized, and X-ray diffraction data were collected. Data collection and refinement statistics are shown in Table 2. The EIIA^ΔHA^ crystal belongs to the *P*1 group with unit cell dimensions of *a* = 52.3, *b* = 53.8, and *c* = 94.9 Å, and α = 91.1, β = 90.0, and *γ* = 61.0°. The final model, containing six molecules in an asymmetric unit, was refined to an *R*_work_ of 20.8% up to a resolution of 1.8 Å. Ramachandran plot analysis indicated 99.0% of residues in the favored regions and 1.00% of residues in the additional allowed regions. The crystal structure of EIIA^ΔHA^ was determined using molecular replacement with *E*. *coli* PTS EIIA^man^ (PDB ID, 1PDO) for mannose import as the initial model.

**Table 2:** Statistics of EIIA^ΔHA^ for data collection and structure refinement.

	EIIA^ΔHA^
**Data collection**	
Space group	*P*1
Cell dimensions	
*a*, *b*, *c* (Å)	52.3, 53.8, 94.9
α, β, *γ* (°)	91.1, 90.0, 61.0
Resolution (Å)	50.0–1.80 (1.86–1.80) *
*R*_merge_	7.4 (24.3)
*I*/σ (*I*)	26.6 (3.25)
Completeness (%)	96.1 (94.5)
Redundancy	2.1 (2.0)
**Refinement**	
Resolution (Å)	33.8–1.80 (1.82–1.80)
No. reflections	80171 (2274)
*R*_work_/*R*_free_	20.8 (26.4)/24.2 (36.8)
No. atoms	
Protein	6038
Glycerol	66
Water	265
*B*-factor (Å^2^)	
Protein	25.8
Glycerol	38.1
Water	29.2
Root mean square deviations	
Bond lengths (Å)	0.006
Bond angles (°)	1.06
Ramachandran plot (%)	
Favored region	99.0
Allowed region	1.00
Outlier region	0

*Data for the highest resolution shell is shown in parenthesis.

### Crystal structure of EIIA^ΔHA^

**Fig 5:**
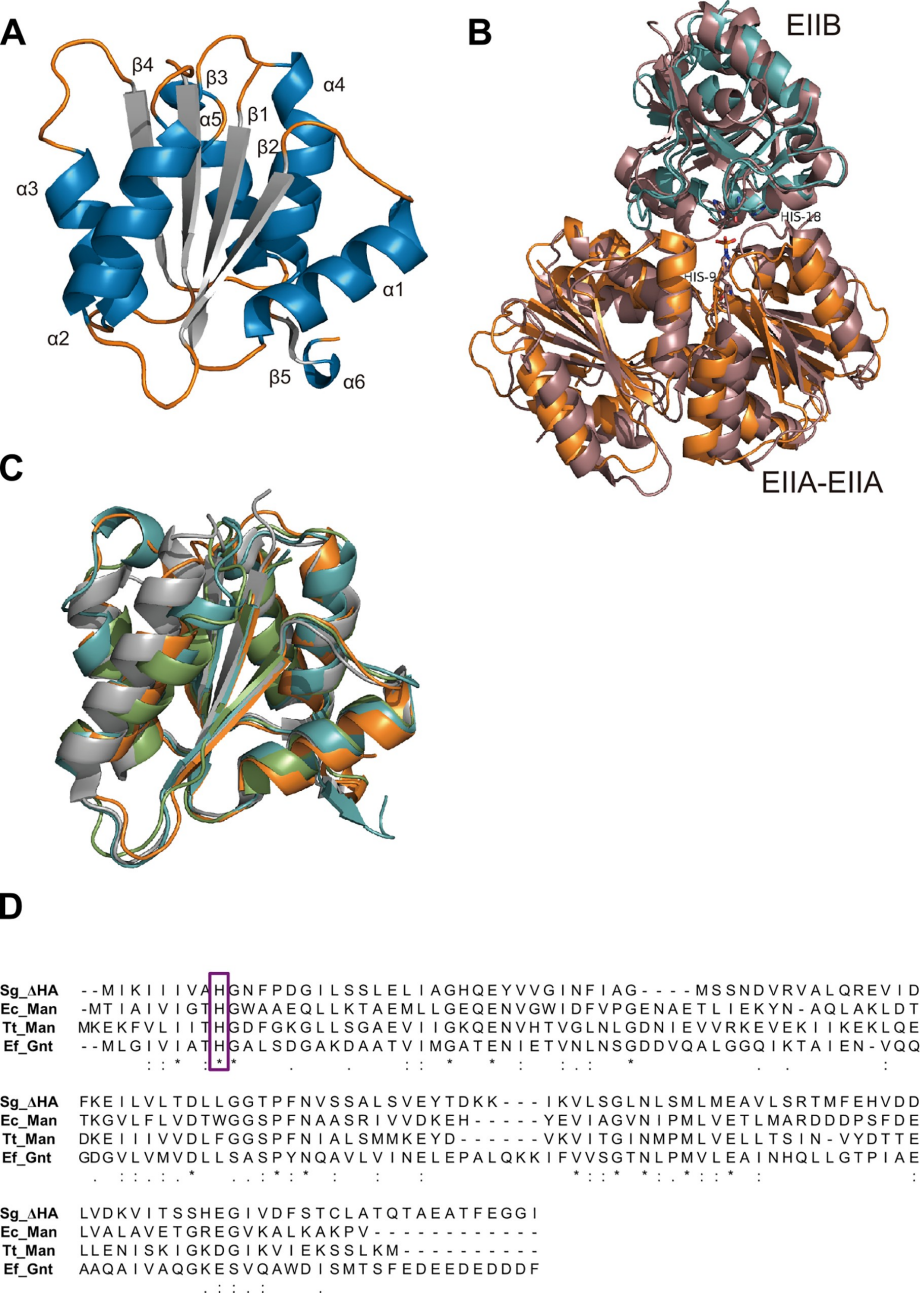
Three-dimensional structure of *S*. *agalactiae* GAG-PTS EIIA^ΔHA^. (A) Overall structure of *S*. *agalactiae* EIIA^ΔHA^. Blue, α-helices; gray, β-strands; and orange, loops. (B) Superimposition of *S*. *agalactiae* EIIA^ΔHA^-EIIB^ΔHA^ and *E*. *coli* EIIA^Man^-EIIB^Man^ complexes. Orange, *S*. *agalactiae* EIIA^ΔHA^; cyan, *S*. *agalactiae* EIIB^ΔHA^ (modeling); dark pink, *E*. *coli* EIIA^Man^-EIIB^Man^ complex. (C) Homologue proteins of *S*. *agalactiae* EIIA^ΔHA^. Orange, *S*. *agalactiae* EIIA^ΔHA^ (Sg_ΔHA); light green, *E*. *coli* mannose EIIA^Man^ (Ec_Man); gray, *T*.*tengcongensis* mannose EIIA^Man^ (Tt_Man); and cyan, *E*. *faecalis* gluconate EIIA^Gnt^ (Ef_Gnt). (D) Alignment of primary structure (EIIA).

EIIA^ΔHA^ consists of 144 residues, although the 14 residues (Leu131–Ile144) of the C-terminal could not be assigned due to their structural flexibility. With respect to the secondary structure of EIIA^ΔHA^, α-helices, β-sheets, and loops constitute 41.0%, 17.0%, and 42.0%, respectively. EIIA^ΔHA^ is composed of six α-helices (α1, Phe12–Ala24; α2, Ser41–Val52; α3, Thr68–Leu76; α4, Leu93–Met105; α5, Asp110–Glu122; and α6, Phe127–Thr129), five β-strands (β1, Lys3–His9; β2, Val30–Phe35; β3, Glu57–Thr62; β4, Lys84–Ser89; and β5, Val125–Asp126), and ten loops (L1, Met1–Ile2; L2, Gly10–Asn11; L3, Gly25–Tyr29; L4, Ile36–Ser40; Ile53–Lys56; L5, Asp63–Gly67; L6, Ser77–Lys83; L7, Gly90–Asn92; L8, Phe106–Val109; L9, Gly123–Ile124; and L10, Cys130). In the overall structure, a parallel β-sheet containing four β-strands (β1, β2, β3, and β4) is located at the center, and two (α2 and α3) and three α-helices (α1, α4, and α5) are located so they pinch the β-sheet from both sides, resulting in the formation of a Rossman-fold frame (Fig 5A). β1–4 and α1–4 are alternately arranged and α4 is followed by α5 then β5. Gel filtration chromatography suggested that EIIA^ΔHA^ was smaller than a tetramer, and the biological asymmetric unit was shown to be a dimer using *PISA* software [43]. In the dimer, the C-terminal β5s in adjacent monomers are arranged to align with mutual β4s, and added to the parallel β-sheet located at the center of the monomer, forming an antiparallel β-sheet.

### Comparison between EIIA^ΔHA^ and *E*. *coli* HPr-EIIA^Man^/EIIA^Man^-EIIB^Man^ complexes

The three-dimensional structures of *E*. *coli* HPr-EIIA^Man^ and EIIA^Man^-EIIB^Man^ complexes (previously determined using NMR), and EIIA^Man^, have also been found to form a dimer [44, 45]. In HPr-EIIA^Man^ and EIIA^Man^-EIIB^Man^ complexes, His10 of EIIA^Man^ is an important residue in the transfer of a phosphate group from HPr to EIIB^Man^, through EIIA^Man^ [46]. Due to the similarity between the interaction sites of EIIA^Man^ with HPr, and EIIA^Man^ with EIIB^Man^, HPr or EIIB^Man^ must be separated while the other remains bound to EIIA^Man^. The His10 of EIIA^Man^ is also conserved in EIIA^ΔHA^ with His9 (Fig 5B). To compare EIIA^ΔHA^-EIIB^ΔHA^ and EIIA^Man^-EIIB^Man^, the EIIA^ΔHA^ dimer and modeled EIIB^ΔHA^ monomer were superimposed with the EIIA^Man^-EIIB^Man^ complex. The arrangements of the His9 of EIIA^ΔHA^ and His10 of EIIA^Man^ almost corresponded to each other; this was also observed with the His18 of EIIB^ΔHA^ and His18 of EIIB^Man^. His9 of EIIA^ΔHA^ is located at the end of β1 and interacts with Asp63 via hydrogen bonding, and with Phe12, Phe35, Asp63, Gly67, and Pro69 through van der Waals contacts. These amino acid residues are almost conserved in EIIA for mannose (including *E*. *coli* EIIA^Man^). Therefore, His9 of EIIA^ΔHA^ appears to be crucial in the transfer of a phosphate group.

## Discussion

The ability of *S*. *agalactiae* to degrade and assimilate hyaluronan allows the measurement of GAG-PTS import activity using an unsaturated disaccharide derived from hyaluronan degradation. The GAG-PTS import of unsaturated hyaluronan disaccharide in bacterial wild-type cells grown in the absence of hyaluronan was significantly higher than in controls using no substrate or GlcN6P; this indicates that *S*. *agalactiae* incorporates unsaturated hyaluronan disaccharide via a PTS. On the other hand, GAG-PTS mutant cells grown in the absence of hyaluronan were unable to incorporate unsaturated hyaluronan disaccharide. Based on these observations and the fact that GAG-PTS mutant failed to grow on hyaluronan-containing minimal medium, we conclude that the GAG-PTS encoded in the GAG genetic cluster is the sole importer of unsaturated hyaluronan disaccharide in *S*. *agalactiae*.

Surprisingly, the PTS import of cellobiose in bacterial wild-type cells grown in the presence of hyaluronan was 2.3 times higher than that in the absence of hyaluronan. Furthermore, the wild-type and the GAG-PTS mutant grown in the presence of hyaluronan exhibited enhanced GAG-PTS import of unsaturated hyaluronan disaccharide, in comparison with the control. Based on these observations, we hypothesize that hyaluronan present in the cultured medium modifies growing *S*. *agalactiae* cells, and the subsequent toluene-treatment causes leakage of cytoplasmic enzymes such as β-glucosidase and UGL. As a result, cellobiose and unsaturated hyaluronan disaccharide contained in the reaction mixtures are degraded by these enzymes to the constituent monosaccharides (glucose, unsaturated GlcUA, and GlcNAc), and incorporated by another PTS.

While bacterial cells import sugars through various mechanisms such as facilitated diffusion, primary and secondary active transport, and group translocation, the PTS is the major sugar import pathway in many bacterial species [30, 47]. PTS Enzyme II is classified into four families based on its primary structure: (i) the glucose-fructose-lactose family; (ii) the ascorbate-galactitol family; (iii) the mannose family; and (iv) the dihydroxyacetone family [48]. Several characteristic features of the mannose family have been defined. These include the observations that EIIC is a hetero (not a homo)-membrane domain in combination with EIID, an EIIB receives a phosphate group from a histidine rather than a cysteine residue, and various sugars can be used as a substrate. *S*. *agalactiae* EIIA^ΔHA^ showed the most similarity with *E*. *coli* mannose EIIA (PDB ID, 1PDO), *Enterococcus faecalis* gluconate EIIA (PDB ID, 3IPR), and *Thermoanaerobacter tengcongensis* mannose/fructose EIIA (PDB ID, 3LFH), all of which belong to the mannose family; Z-scores, estimated by the Dali program [49], were 20.2, 19.9, and 19.1, respectively (Fig 5C and 5D) (S1 Table). Based on the well-conserved characteristics of the mannose family, the *S*. *agalactiae* GAG-PTS for the import of unsaturated hyaluronan disaccharide appears to be a member of this family. The three-dimensional structures of these enzymes were well superimposed (Fig 5C). *S*. *agalactiae* EIIB^ΔHA^ was homology modeled by the SWISS-MODEL [50] using putative *S*. *pyogenes* EIIB for the import of GalNAc (PDB ID, 3P3V) (sequence identity: 70%) as a template. *S*. *agalactiae* EIIB^ΔHA^ is composed of an antiparallel β-sheet of eight β-strands, and eight α-helices (Fig 5B).

In this study, *S*. *agalactiae* was found to import unsaturated hyaluronan disaccharide through the GAG-PTS encoded in the GAG genetic cluster. Unlike other sulfated GAGs, hyaluronan contains no sulfate groups at the C-6 position of its constituent monosaccharides. Thus, unsaturated hyaluronan disaccharide is a suitable substrate for the GAG-PTS through the transfer of a phosphate group to the C-6 position. We have recently identified a solute-binding protein-dependent ABC transporter in Gram-negative *S*. *moniliformis* that acts as an importer of unsaturated GAG disaccharides [18, 51] (S5 Fig). Bacterial ABC transporters generally receive substrates from solute-binding proteins and incorporate the substrates into the cytoplasm using the energy of ATP hydrolysis [52]. Because the imported substrates of the ABC transporter have no modifications that render them distinct from PTS substrates, *S*. *moniliformis* ABC transporter has been demonstrated to import both sulfated and non-sulfated unsaturated GAG disaccharides derived from chondroitin sulfate and hyaluronan. Furthermore, genes homologous with *S*. *moniliformis* ABC transporter genes are conserved among genomes of several fusobacterial species, which are generally indigenous to animal oral cavities. Fusobacterium probably utilizes the ABC transporter for the assimilation of sulfated GAGs that are abundant in the oral cavities. On the other hand, *S*. *agalactiae*, a pathogen of the hyaluronan-rich vaginal mucosa [53, 54], is thought to utilize the GAG-PTS to assimilate vaginal hyaluronan. Streptococci, and several intestinal probiotics such as *Lactobacillus rhamnosus* and *E*. *faecalis* [55], possess the genes homologous with the GAG-PTS [56], indicating that the import system may be common to the intestinal bacteria that are able to use GAGs. Based on the substrate preference, the intestinal probiotics seem to utilize heparin and/or heparan sulfate-specific PTS while pathogenic streptococcal GAG-PTS functions to import unsaturated hyaluronan disaccharide. Therefore, the GAG-PTS specific for hyaluronan may become a target for development of novel drugs against infectious diseases by pathogenic streptococci.

In conclusion, this is the first report that *S*. *agalactiae* GAG-PTS encoded in the GAG genetic cluster is the importer of non-sulfated unsaturated hyaluronan disaccharides distinct from sulfated GAG-fragments.

## Supporting information

S1 Table Structure similarity of *S*. *agalactiae* EIIA^ΔHA^. (DOCX)

S1 Fig Structural formulas of GAGs. (DOCX)

S2 Fig Metabolic pathways of ΔGlcUA and GlcUA. (DOCX)

S3 Fig Gene disruption. (DOCX)

S4 Fig Growth of *S*. *agalactiae* in nutrient medium in the presence or absence of hyaluronan. (DOCX)

S5 Fig Gram-positive *Streptococcus* PTS and Gram-negative *Streptobacillus* ABC transporter. (DOCX)
