# Supplementary material for: Streptococcal phosphotransferase system imports unsaturated hyaluronan disaccharide derived from host extracellular matrices
Source: PLoS One. 2019 Nov 7;14(11):e0224753. doi: 10.1371/journal.pone.0224753 (PMC6837340; doi:10.1371/journal.pone.0224753)
Supplement: S1 Table — (DOCX) [file pone.0224753.s001.docx]

**S1 Table. Structure similarity of *S. agalactiae* EIIA^ΔHA^**

| Annotation | PDB ID | Z-score |
| --- | --- | --- |
| *E. coli* mannose EIIA | 1PDO | 20.2 |
| *E. faecalis* gluconate EIIA | 3IPR | 19.9 |
| *T. tengcongensis* mannose/fructose EIIA | 3LFH | 19.1 |
| *E. faecalis* mannose/sorbose EIIA | 3BED | 19.0 |
| *E. faecalis* unknown protein | 3GDW | 15.4 |
| *E. faecalis* unknown protein | 3B48 | 12.2 |
| *Ruminococcus gnavus* unknown protein | 3JR7 | 11.2 |
| *Staphylococcus aureus* fatty acid kinase | 5UTO | 10.8 |
| *Corynebacterium glutamicum* unknown protein | 3EGL | 10.2 |
| *S. pyogenes* unknown protein | 2G7Z | 9.7 |
