# Supplementary figures and images for: Streptococcal phosphotransferase system imports unsaturated hyaluronan disaccharide derived from host extracellular matrices
Source: PLoS One. 2019 Nov 7;14(11):e0224753. doi: 10.1371/journal.pone.0224753 (PMC6837340; doi:10.1371/journal.pone.0224753)

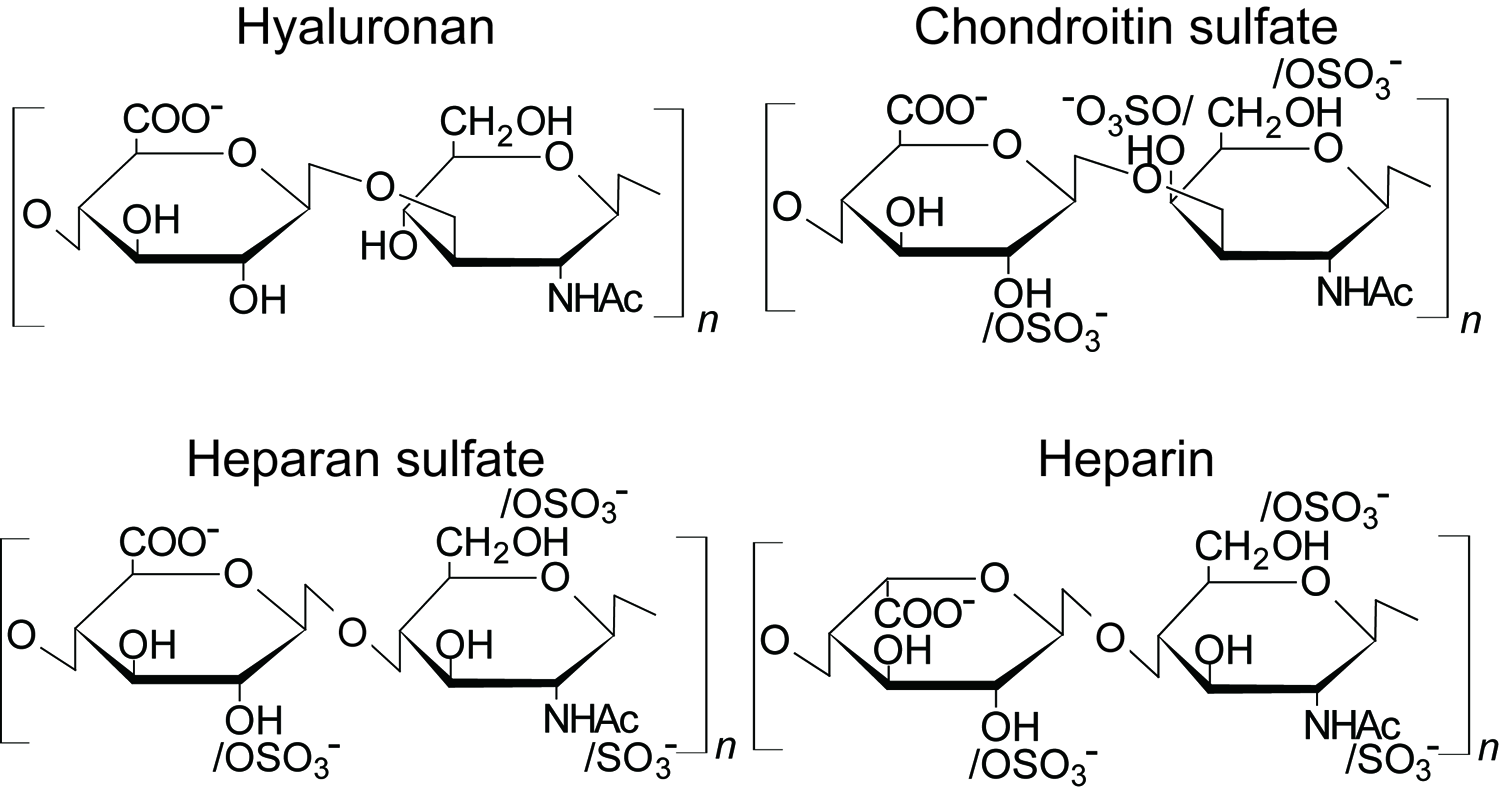


**S1 Fig. Structural formulas of GAGs**

Supplement: S1 Fig — (DOCX) [file pone.0224753.s002.docx]
