# Supplementary material for: Streptococcal phosphotransferase system imports unsaturated hyaluronan disaccharide derived from host extracellular matrices
Source: PLoS One. 2019 Nov 7;14(11):e0224753. doi: 10.1371/journal.pone.0224753 (PMC6837340; doi:10.1371/journal.pone.0224753)
Supplement: S2 Fig — (DOCX) [file pone.0224753.s003.docx]

**
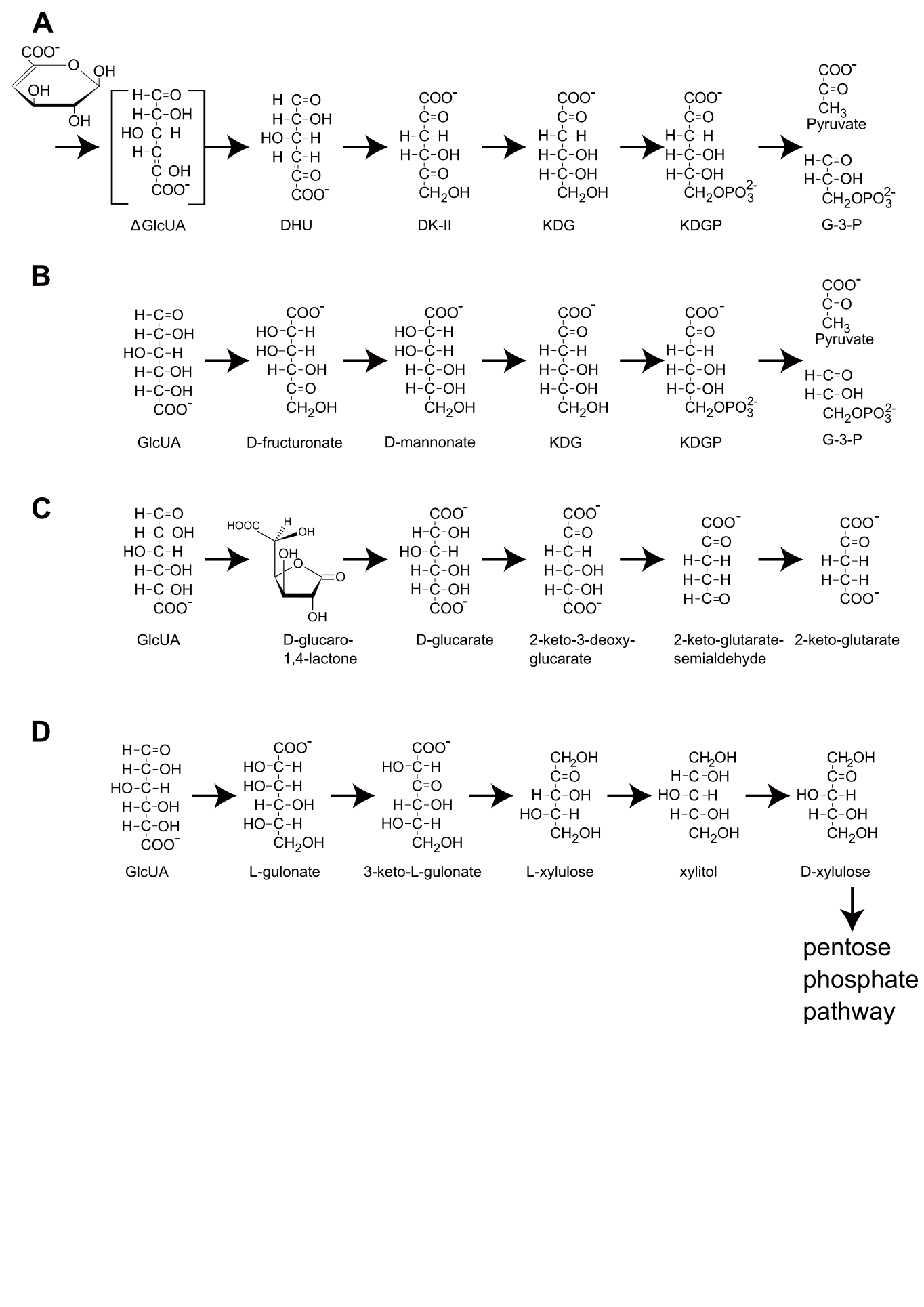
**

**S2 Fig. Metabolic pathways of ΔGlcUA and GlcUA** [16, 17]**.**

Bacterial metabolic pathway of ΔGlcUA produced by GAG lyases (A). Bacterial metabolic pathways of GlcUA produced by GAG hydrolases with isomerase (B) and oxidation (C). Mammal metabolic pathway of GlcUA (D).
