# Supplementary material for: Streptococcal phosphotransferase system imports unsaturated hyaluronan disaccharide derived from host extracellular matrices
Source: PLoS One. 2019 Nov 7;14(11):e0224753. doi: 10.1371/journal.pone.0224753 (PMC6837340; doi:10.1371/journal.pone.0224753)
Supplement: S3 Fig — (DOCX) [file pone.0224753.s004.docx]

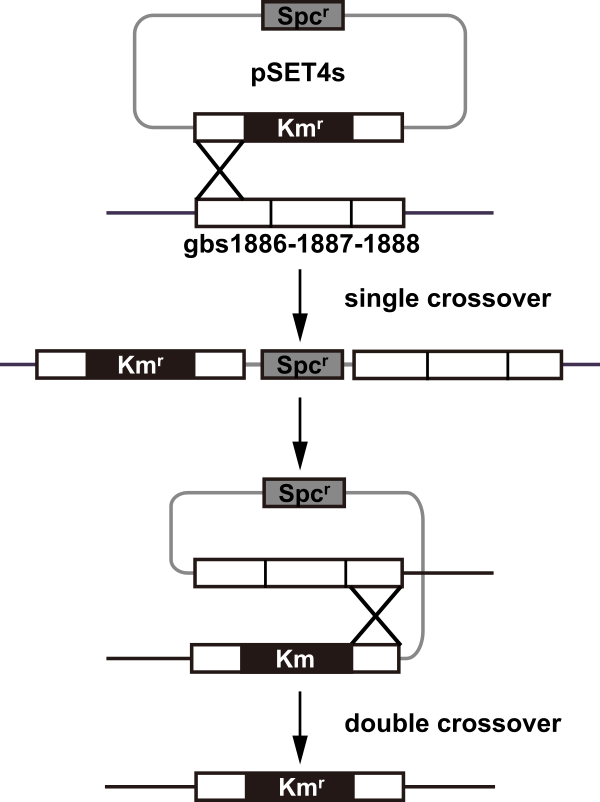


**S3 Fig. Gene disruption.**

Construct of the PTS mutant. The gbs1886-1887-1888 operon gene in the pSET4s plasmid was disrupted by the insertion of Km^r^. The pSET4s-gbs1886-1887-1888::Km^r^ plasmid was introduced into the streptococcal cells. A double crossover mutant was obtained by homologous recombination.
