# Supplementary material for: Streptococcal phosphotransferase system imports unsaturated hyaluronan disaccharide derived from host extracellular matrices
Source: PLoS One. 2019 Nov 7;14(11):e0224753. doi: 10.1371/journal.pone.0224753 (PMC6837340; doi:10.1371/journal.pone.0224753)
Supplement: S4 Fig — (DOCX) [file pone.0224753.s005.docx]

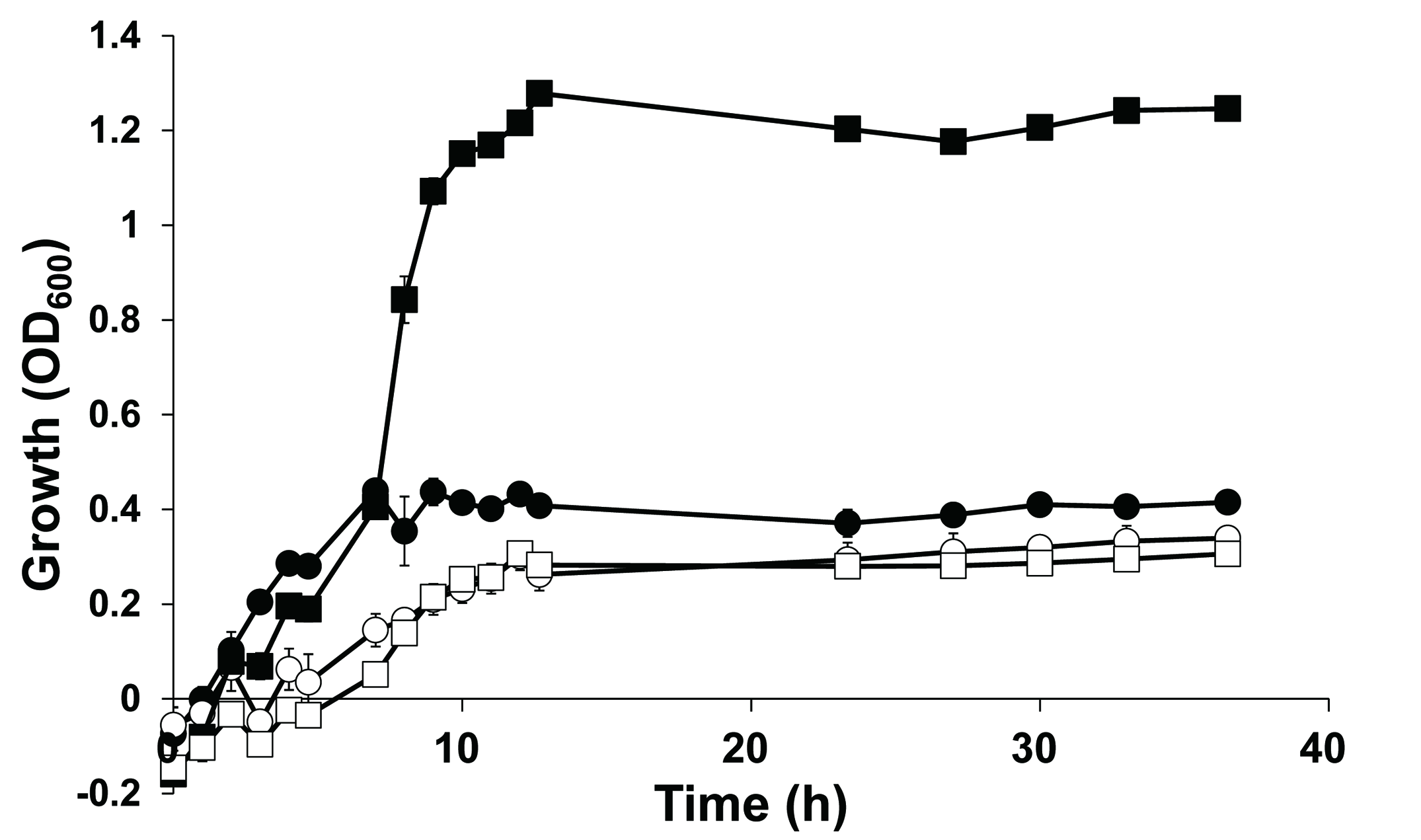


**S4 Fig. Growth of *S. agalactiae* in nutrient medium in the presence or absence of hyaluronan.**

Wild-type (closed) and the PTS mutant (open) in nutrient medium in the presence (square) or absence (circle) of hyaluronan. Each measurement represents the mean of three individual experiments (means ± standard deviations).
