# Supplementary material for: Streptococcal phosphotransferase system imports unsaturated hyaluronan disaccharide derived from host extracellular matrices
Source: PLoS One. 2019 Nov 7;14(11):e0224753. doi: 10.1371/journal.pone.0224753 (PMC6837340; doi:10.1371/journal.pone.0224753)
Supplement: S5 Fig — (DOCX) [file pone.0224753.s006.docx]

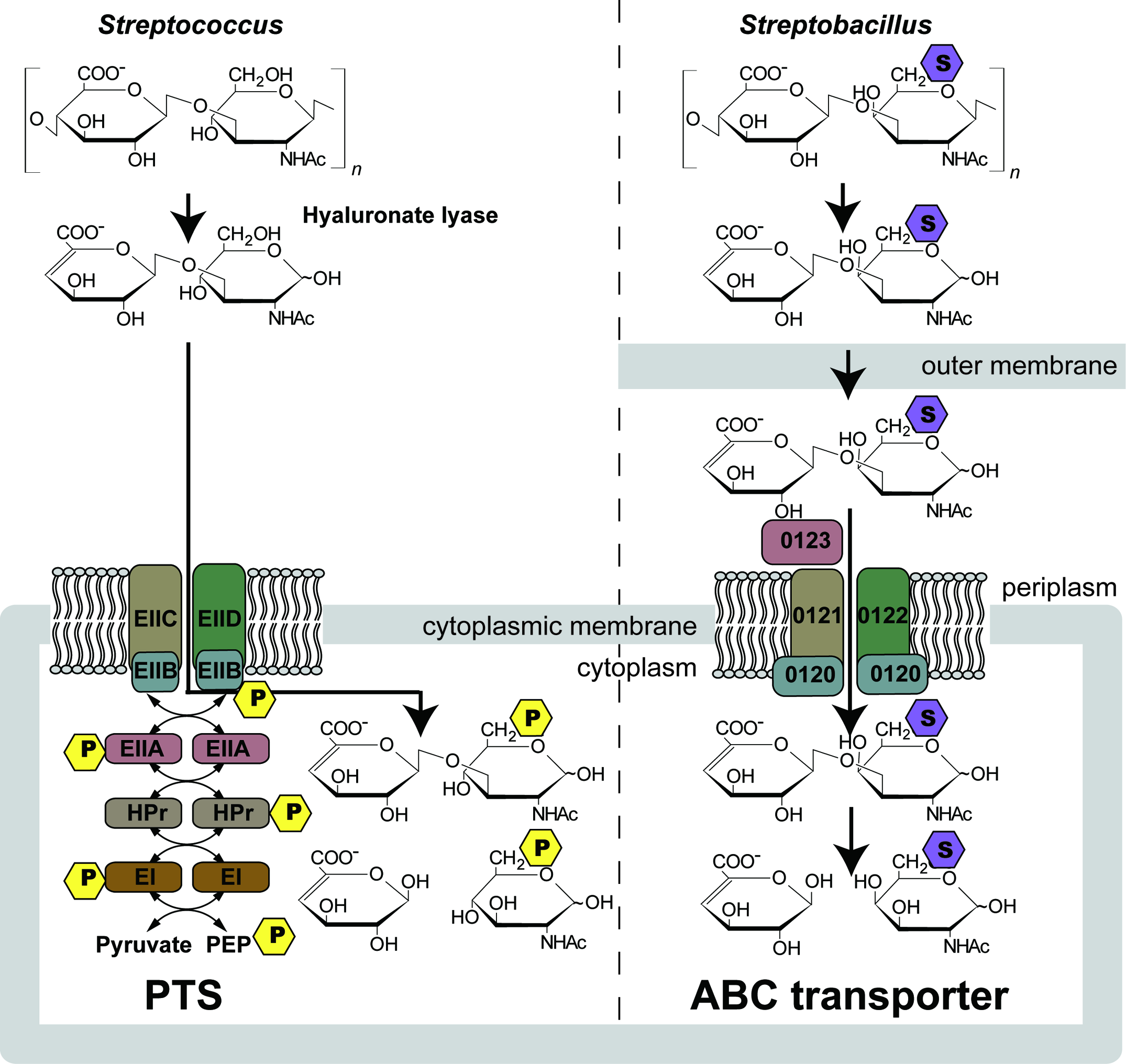


**S5 Fig. Gram-positive *Streptococcus* PTS and Gram-negative *Streptobacillus* ABC transporter.**

In *Streptococcus*, hyaluronan is depolymerized to unsaturated hyaluronan disaccharide by cell-surface hyaluronate lyase. Unsaturated hyaluronan disaccharides are incorporated into the cytoplasm by the PTS with the phosphorylation at the C-6 position. Imported disaccharides with the phosphate group are degraded to monosaccharides by cytoplasmic UGL (left). In *Streptobacillus*, depolymerized GAG disaccharides with sulfate groups are incorporated into the cytoplasm by the ABC transporter (Smon0121-Smon0122/Smon0120-Smon0120) through the periplasmic solute-binding protein (Smon0123) without any substrate modification (right).
